# Infection and Mechanical Complications Are Risk Factors for New Diagnosis of a Mental Health Disorder After Total Joint Arthroplasty

**DOI:** 10.1016/j.artd.2021.05.019

**Published:** 2021-06-21

**Authors:** Andrew Michael Figoni, Gopal R. Lalchandani, Alexander R. Markes, David Sing, Erik Nathan Hansen

**Affiliations:** a Department of Orthopaedic Surgery, University of California San Francisco, San Francisco, CA, USA; b Department of Orthopaedic Surgery, Boston University Medical Center, Boston, MA, USA

**Keywords:** Mental health disorder, Prosthetic joint infection, Total hip arthroplasty, Total knee arthroplasty, Revision arthroplasty

## Abstract

**Background:**

Prior studies have demonstrated that depression is an independent risk factor for developing a prosthetic joint infection (PJI) after total joint arthroplasty (TJA). To our knowledge, there is no literature examining whether PJI or aseptic failure increases the risk of developing a new mental health diagnosis.

**Methods:**

PearlDiver Patient Database (Colorado Springs, CO) was used to identify 80,826 patients who underwent TJA without a pre-existing diagnosis of depression, anxiety, a stress and/or adjustment disorder, and/or current use of a selective serotonin reuptake inhibitor within the year prior to surgery. The odds of developing a new mental health issue or being prescribed a selective serotonin reuptake inhibitor within 1 year of an uncomplicated TJA was compared to those who developed PJI or mechanical failure within 90 days after TJA as well as to those who subsequently underwent revision surgery within 30 days of either complication using Fisher’s exact test and Baptista-Pike.

**Results:**

A total of 6474 (8%) patients were diagnosed with a new mental health issue after TJA. PJI or mechanical failure led to significantly higher odds of new diagnoses with an odds ratio of 1.67 (95% confidence interval = 1.26, 2.22) and 1.57 (1.24, 2.00), respectively. Undergoing revision surgery for PJI or mechanical failure increased the odds of developing a new mental health diagnosis to 2.10 (1.29, 3.42) and 2.24 (1.36, 3.72), respectively. There was no significant difference comparing those who developed PJI vs those who sustained mechanical complications.

**Conclusion:**

Patients who sustain complications after TJA are at increased odds of receiving a new mental health diagnosis, an effect further amplified if revision surgery is required.

## Introduction

Mental health disorders are common, and are diagnosed with increasing frequency in the United States [1]. Pre-existing mental health disorders are a risk factor for the development of prosthetic joint infection (PJI) after total hip and knee arthroplasty (THA and TKA). This correlation has been demonstrated in multiple cohort, database, and meta-analysis studies [[2], [3], [4], [5]], with some authors advocating for routine preoperative screening for depression and other psychiatric conditions.

Literature on the consequences of PJI and aseptic failure after total joint arthroplasty (TJA) has been well documented. These complications are associated with poor clinical outcomes, often requiring staged management and leaving patients with poor overall health-related quality of life [3,6,7]. Furthermore, compared with aseptic revisions, revisions for PJI have been shown to have a significantly higher risk of major postoperative complications including death, sepsis, non-home discharge, readmissions, and prolonged hospital length of stay [8]. However, to our knowledge, there are no published studies examining the relative risk of developing new mental health diagnoses after diagnosis of PJI or mechanical failure. In this study, the odds of new diagnoses of depression, anxiety, stress disorder, and/or adjustment disorder after PJI or mechanical prosthetic complication after TJA was compared to patients who underwent uncomplicated THA or TKA. We hypothesized that patients who developed a PJI or mechanical complication were at increased odds of subsequent mental health disorders compared with patients who did not develop these complications after TJA.

## Material and methods

This analysis used the PearlDiver Patient Records Database (Colorado Springs, CO), which is a retrospective nationwide insurance billing database of over 25 million patients. The records in the PearlDiver Patient Records Database are acquired from Humana’s (Louisville, KY) claims database, deidentified, and released commercially for research purposes. Humana is a private insurance company that offers both commercial and Medicare advantage plans. Claims in the PearlDiver database are from patients enrolled in either of Humana’s commercial or Medicare advantage plans between 2006 and 2014.

Current Procedural Terminology, International Classification of Diseases (ICD)-9 and ICD-10 codes, and pharmacy claims data were used to identify our cohorts as detailed in Appendix 1. We identified patients who underwent a THA or TKA between 2006 and 2014. Patients were included if they were insured by the same carrier for at least 1 year before and after their index surgery to ensure continuity of data. Patients were excluded if they carried a mental health diagnosis or had a prescription for a selective serotonin reuptake inhibitor (SSRI) medication in the year before surgery. We then defined our PJI cohort as infections occurring within 90 days of their index surgery as defined by ICD-9 or ICD-10 codes. Similarly, we defined a mechanical failure cohort as patients who had ICD-9 or ICD-10 diagnoses consistent with mechanical failure within 90 days of their index surgery.

The development of a new mental health diagnosis within the subsequent year after index surgery was defined by the presence of a new ICD-9 or ICD-10 code for depression, anxiety, stress, and/or adjustment disorder and/or filling a new prescription for an SSRI medication. SSRIs were the only pharmacotherapy evaluated, as they are the most commonly used first-line medication in the treatment of depression [9]. In addition, other classes of medications used to treat depression such as serotonin-norepinephrine reuptake inhibitors or tricyclic antidepressants are also commonly used for treatment of other conditions such chronic and/or neuropathic pain; thus, we felt limiting our analysis to SSRIs alone would be most specific for concomitant psychiatric pathology. We then compared odds of developing a new mental health diagnosis between patients undergoing primary uncomplicated TJA, those who developed PJI, and those who developed mechanical failure. For both PJI and aseptic failures, we used 11 different Current Procedural Terminology codes for revision arthroplasty procedures (Appendix 1) to analyze whether undergoing revision surgery within the first 30 days after the diagnosis of a complication affected the odds of new mental health diagnoses further. The decision to analyze acute complications within 90 days of the index surgery and revision surgery within the 30 days after diagnosis of that complication was a choice of the authors to best maintain the temporal relationship of surgery followed by PJI or aseptic failure and then subsequent diagnosis of mental health disorder. It was our feeling that broadening to include more chronic PJI may decrease the accuracy and the reliability of this temporal relationship. In addition, the authors felt only analyzing revision surgery within 30 days after the diagnosis of the complication, as opposed to within 90 days or further from the complication, would ensure that the procedure code listed was in fact for the joint operated on during the index procedure and the resulting complication. The authors posited that the chances of a patient undergoing a procedure on another joint followed by a revision surgery in this timeframe was sufficiently low, thus further improving the accuracy of our search.

Fisher’s exact test was used to determine statistical significance, and the Baptista-Pike test was used to determine the odds ratio (OR) and confidence intervals (CI). A significant *P* value was set to 0.05. Data management was performed using Microsoft Excel (Microsoft, Redmond, WA). All statistical analyses were performed using Prism 8 (GraphPad, San Diego, CA).

## Results

**Figure 1:**
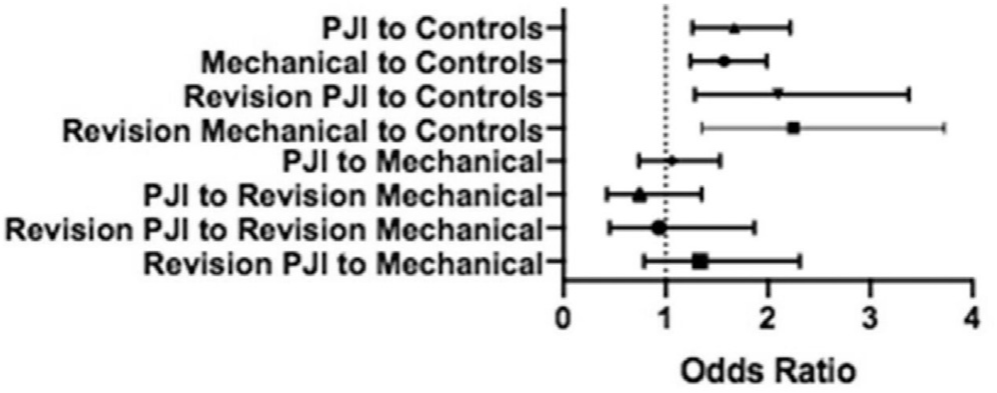
Odds ratios of cohorts compared in analysis.

We identified 235,208 patients who underwent primary unilateral TJA from 2006 to 2014; of which, 112,965 patients were insured in the year before and after their index surgery. Of those who were insured, 32,139 (28%) were excluded because of the presence of a pre-existing mental health diagnosis and/or having filled a prescription for SSRI within a year of the index surgery. Of the remaining 80,826 insured patients undergoing primary TJA without a previous mental health diagnosis or subsequent complication, 8% (6,474) developed a new mental health diagnosis in the first postoperative year (Table 1). In comparison, a significantly higher percentage of new psychiatric diagnoses was seen in patients who developed PJI within 90 days of surgery (12.7% [55/433], *P* = .0007) and in those with mechanical complications (12% [78/648] *P* = .0004). We calculated an OR of 1.67 (95% CI = 1.26 to 2.22, *P* = .0004) of developing a mental health disorder within 1 year in patients diagnosed with an acute PJI when compared to patients who did not develop any complication (Fig. 1). Of those who then underwent revision surgery within 30 days of their PJI diagnosis, the percentage increased to 15.5% (19/123 *P* = .0067), and the OR increased to 2.10 (95% CI = 1.29 to 3.42, *P* = .003). For those with a mechanical complication within 90 days, the OR for developing a new psychiatric diagnosis was 1.57 (95% CI = 1.24 to 2.00, *P* = .0002), which increased to 2.24 (95% CI = 1.36 to 3.72, *P* = .0017) for those who subsequently underwent revision surgery within 30 days of their mechanical complication diagnosis. The percentage of new mental health disorders increased to 16.4% (18/110 *P* = .0039). There was no significant difference when comparing within the groups who were diagnosed with either a PJI or mechanical complication.

**Table 1:** Percentages of patients with new mental health diagnoses.

Study subgroups	New mental disorder diagnosis	Patients meeting inclusion criteria	Percentages	*P*^a^
Controls	6474	80,826	8.01	
PJI within 90 d	55	433	12.70	.0007
Revision surgery within 30 d of the PJI diagnosis	19	123	15.45	.0067
Mechanical complications within 90 d	78	648	12.04	.004
Revision surgery within 30 d of the mechanical complications	18	110	16.36	.0039

a *P* value calculated comparing corresponding group to controls.

## Discussion

Patients who develop complications after TJA are at increased odds of receiving a new mental health diagnosis, an effect further amplified if revision surgery is required. To our knowledge, this is the first study examining the psychological impact of developing complications after TJA.

Prevalence of pre-existing mental health disorders in patients undergoing primary TKA studied in the literature varies between 3% and 14.5% [[2], [3], [4], [5]]. In our cohort of 112,965 patients who were insured 1 year before surgery and underwent primary TJA, we found an overall prevalence for pre-existing mental health diagnosis or prescription for SSRI of 28%. Although greater than previously reported, we posit that our inclusion of patients with a broader range of mental health diagnoses including those who filled a prescription for an SSRI may account for this difference. In the largest of the previously referenced studies, Klement et al. identified over 1.4 million patients who underwent primary TKA from 2005 to 2011 using 100% of the Medicare claims database [4]. They defined a cohort with pre-existing mental health conditions of approximately 200,000 patients leading to a prevalence of 14.5%. Although lower than our prevalence, their cohort only included patients with bipolar disorder (1.5%), depression (12.5%), and schizophrenia (0.5%) [4].

Pre-existing mental health diagnosis as a risk factor for PJI has also been well-studied [[2], [3], [4], [5],^10^,^11^]. Browne et al. found a 10% prevalence of pre-existing depression in 500,000 patients who underwent TJA and noted that patients with depression had an OR of 1.33 of developing postoperative infection compared with those without a diagnosis [5]. Similarly, in a multicenter case-control study, Bozic et al. used multivariable regression to analyze significant risk factors for PJI after THA [3]. Among 18 other comorbid conditions, depression was found to be most significantly associated with increased risk of PJI with an adjusted hazard ratio of 1.96 [3].

While mental health diagnoses affect development of PJI, analysis of the psychological burden of arthroplasty and its associated complications is lacking. Development of mental health disorders has been demonstrated after cardiac bypass surgery, bone marrow transplant, and abdominal aortic aneurysm repair [[12], [13], [14]]. Doerfler et al. showed up to 19% of patients receiving bone marrow transplants for breast cancer develop posttraumatic stress disorder, while Liberzon et al. demonstrated a 32% incidence of new diagnosis of depression and/or posttraumatic stress in intensive care patients after abdominal aortic surgery [12,14].

In our cohort, we found that 1 in 12 patients who undergo TJA was given a new diagnosis of mental health disorder. Onset of depression after surgical complications is most likely multifactorial, but may be associated with thoughts that surgical complications are seen as an unsatisfying failure, both from the surgeon and patient's standpoint. Numerous studies have reported on satisfaction rates after TJA varying between 77% and 89%, with complications being associated with lower satisfaction rates [[15], [16], [17], [18], [19], [20], [21], [22], [23], [24], [25]]. Bourne et al. analyzed 1703 primary TKAs and found that patients who sustained a postoperative complication requiring readmission were 1.9 times more likely to be “unsatisfied” with the procedure than a patient who underwent uncomplicated TKA [15]. Similarly, our study demonstrated a greater than 2 times odds of developing a new mental health disorder after TJA with revision surgery for PJI or aseptic failure. While no study has associated development of depression after TJA with patient-reported “satisfaction” from the procedure, it is the hypothesis of the authors that there is likely some association between the 2. Qualitative studies on patients after TKA demonstrate that, even in the absence of complications, patients are frequently burdened with thoughts that postoperative changes they are feeling are complications from surgery [25]. The psychological burden of having sustained these feared complications, or worse needing to undergo revision surgery for these complications, as demonstrated in our analysis, plays a significant role in the satisfaction and mental well-being of our patients and should be pre-emptively evaluated in all patients after diagnosis of PJI or aseptic failure.

As suggested by Ghoneim and O'Hara in their review of surgical complications and depression, even in fairly busy surgical practices, it is relatively easy to screen for depression [26]. They advocate for the use of a preoperative screening tool such as the Patient Health Questionnaire-9 which is a validated 9-item survey frequently used in the clinical setting to screen for depression [27]. Although they advocate for its use preoperatively, it can similarly be used postoperatively for all patients diagnosed with septic or aseptic complications, particularly those scheduled to undergo revision surgery. To our knowledge, there are no clinical studies evaluating outcomes of automated screening of all patients with surgical complications after TJA. Given the significant impact these complications and any subsequent mental health disorders have on our patient population, it is a potentially promising avenue for future inquiry.

In addition, although previous studies have shown worse outcomes when comparing PJI to aseptic complications [7,8], our study found no difference between rates of new diagnosis between patients who suffered aseptic complications vs. infection, as well as between a subset of these 2 cohorts who subsequently went on to revision surgery. The authors posit one explanation for this is the inclusion of only acute PJI in the analysis. Acute PJI would more readily be managed with a single secondary surgery than PJI occurring after 90 days. For which multistage surgeries are considered the standard of care. Future studies are needed to further elucidate potential differences between the psychological ramifications of acute vs delayed-onset complications potentially requiring a more complex and burdensome management course.

While one of the advantages of this study is the vast patient population pulled from all regions of the country, there are limitations to relying on a large administrative database. The PearlDiver database did not allow us to extensively evaluate medical history or comorbidities because of the relatively small numbers of patients in some of our subgroups. Specifically, when attempting to breakdown demographics such as age, gender, ethnicity, etc. or comorbidities using the Elixhauser Comorbidity Index (ECI), PearlDiver will not result the specific number of patients if there are fewer than 10 patients in that subgroup. For example, if fewer than 10 patients in our subgroup of patients who developed PJI and underwent revision surgery had an ECI of 6, PearlDiver reports the number of patients in that group as “-1” and not the actual number of patients in that group. Despite this limitation, PearlDiver will still result the total number of patients, the average ECI, the median ECI, and the standard deviation for all patients in the group who were diagnosed with a PJI and underwent revision surgery. This precluded our ability to perform univariate or multivariate analysis to isolate for confounding variables. We attempted to minimize confounding pre-existing mental health conditions by excluding patients with a diagnosis in the year leading up to their surgery. However, in doing so, we excluded a significant number of patients. In addition, the analysis hinges heavily on the accuracy of patient coding. While miscoding is not an uncommon occurrence, in theory, the large patient sample size in our cohort should reduce the effect of erroneous claims coding [28].

## Conclusions

Using a publicly available database of privately insured patients, we confirmed our anecdotal suspicion that patients diagnosed with a PJI or a mechanical complication shortly after a TJA are at increased odds of developing a newly diagnosed mental health disorder or receiving a prescription for an SSRI within 1 year of their complication. Surgeons may consider a multidisciplinary approach to the overall care of their patients suffering from the significant psychological burden of these complications. In addition, moving forward, prospective studies on the mental health outcomes of patients suffering complications should be investigated.

## Conflicts of interest

The authors declare that they have no known competing financial interests or personal relationships that could have appeared to influence the work reported in this article.

## Appendix

### Current Procedural Terminology (CPT) for Total Hip Arthroplasty (THA) & Total Knee Arthroplasty (TKA)

TKA – 27,447; THA - 27,130

### CPT codes for revision procedures

27,090, 27,091, 27,130, 27,132, 27,134, 27,137, 27,138, 27,447, 27,486, 27,487, 27,488

### International Classification of Diseases (ICD) -9 and -10 codes for mental health diagnoses

Major Depressive Disorder

ICD-9 = {296.21. 296.31, 296.22, 296.32, 296.23, 296.33, 296.24, 296.34, 296.2, 296.3]

ICD-10 = {F32.0, F32.1, F33.1, F32.2, F33.2, F32.3, F33.3, F32.9, F33.9}

General Anxiety Disorder

ICD-9 = {300.02, 300.09, 300}

ICD-10 = {F41.1, F41.3, F41.8, F41.9}

Stress Disorders

ICD-9 = {308, 309.81, 309.89, 308.3, 308.9}

ICD-10 = {F43.0, F43.1, F43.2, F43.8, F43.9}

### ICD -9 and -10 codes for prosthetic joint infection (PJI)

ICD-9 = {996.66}

ICD-10 = {T84.53XA, T84.54XA, T84.53XD, T84.54XD, T84.53XS, T84.54XS, T84.51XA, T84.52XA, T84.51XD, T84.52XD, T84.51XS, T84.52XS}

### ICD -9 and -10 codes for aseptic complications

ICD9 = {996.40, 996.41, 996.42, 996.43, -996.44, 996.45, 996.46, 996.47}

ICD10 = {T84.012A, T84.012D, T84.012S, T84.013A, T84.013D, T84.013S, T84.022A, T84.022D, T84.022S, T84.023A, T84.023D, T84.023S, T84.032A, T84.032D, T84.032S, T84.033A, T84.033D, T84.033S, T84.042A, T84.042D, T84.042S, T84.043A, T84.043D, T84.043S, T84.052A, T84.052D, T84.052S, T84.053A, T84.053D, T84.053S, T84.062A, T84.062D, T84.062S, T84.063A, T84.063D, T84.063S, T84.092A, T84.092D, T84.092S, T84.093A, T84.093D, T84.093S, T84.010A, T84.010D, T84.010S, T84.011A, T84.011D, T84.011S, T84.020A, T84.020D, T84.020S, T84.021A, T84.021D, T84.021S, T84.030A, T84.030D, T84.030S, T84.031A, T84.031D, T84.031S, T84.040A, T84.040D, T84.040S, T84.041A, T84.041D, T84.041S, T84.050A, T84.050D, T84.050S, T84.051A, T84.051D, T84.051S, T84.060A, T84.060D, T84.060S, T84.061A, T84.061D, T84.061S, T84.090A, T84.090D, T84.090S, T84.091A, T84.091D, T84.091S}

## Appendix A Supplementary data

Conflict of Interest Statement for Figoni

Conflict of Interest Statement for Hansen

Conflict of Interest Statement for Lalchandani

Conflict of Interest Statement for Markes

Conflict of Interest Statement for Sing
